# An experimental point of view on hydration/solvation in halophilic proteins

**DOI:** 10.3389/fmicb.2014.00066

**Published:** 2014-02-21

**Authors:** Romain Talon, Nicolas Coquelle, Dominique Madern, Eric Girard

**Affiliations:** ^1^Institut de Biologie Structurale, Université Grenoble AlpesGrenoble, France; ^2^CEA, DSV, Institut de Biologie StructuraleGrenoble, France; ^3^Institut de Biologie Structurale, Centre National de la Recherche ScientifiqueGrenoble, France

**Keywords:** halophilic, solvation, hydration, water pentagon, malate dehydrogenase, acidic proteins, adaptation, *salinibacter*

## Abstract

Protein-solvent interactions govern the behaviors of proteins isolated from extreme halophiles. In this work, we compared the solvent envelopes of two orthologous tetrameric malate dehydrogenases (MalDHs) from halophilic and non-halophilic bacteria. The crystal structure of the MalDH from the non-halophilic bacterium *Chloroflexus aurantiacus* (*Ca* MalDH) solved, *de novo*, at 1.7 Å resolution exhibits numerous water molecules in its solvation shell. We observed that a large number of these water molecules are arranged in pentagonal polygons in the first hydration shell of *Ca* MalDH. Some of them are clustered in large networks, which cover non-polar amino acid surface. The crystal structure of MalDH from the extreme halophilic bacterium *Salinibacter ruber* (*Sr*) solved at 1.55 Å resolution shows that its surface is strongly enriched in acidic amino acids. The structural comparison of these two models is the first direct observation of the relative impact of acidic surface enrichment on the water structure organization between a halophilic protein and its non-adapted counterpart. The data show that surface acidic amino acids disrupt pentagonal water networks in the hydration shell. These crystallographic observations are discussed with respect to halophilic protein behaviors in solution

## Introduction

*Salinibacter ruber* (*Sr*) is a halophilic bacterium that was isolated from saltern crystallizer ponds in Spain (Antón et al., 2002). In contrast to most bacterial species that equilibrate osmotic pressure with compatible solute, *S. ruber* accumulates high KCl concentration within its cytoplasm, an adaptive strategy similar to that of haloarchaea (*Halobacteriaceae*) (Oren, 2002). *S. ruber* genome sequence has revealed some interesting characteristics related to haloadaption: numerous lateral gene transfers from haloarchaea and a mean *pI*-value of 5.2 of its whole proteome (Mongodin et al., 2005). This proteomic pI shift toward low values, which is typical in haloarchaea, is the consequence of an enrichment of Asp and Glu residues and is considered an adaptive signature of proteins facing high salt concentration (Oren, 2013). However this explanation has been recently challenged by the characterization of a bacterium (*Halorodospira*) that does not accumulate high KCl concentration in its cytoplasm and has nonetheless a high acidic proteome (Deole et al., 2013). Among the few cytoplasmic enzymes isolated from *S. ruber* (Bonete et al., 2003; Madern and Zaccai, 2004), the tetrameric malate dehydrogenase (MalDH) remains the most extensively characterized, at the biochemical and structural level (Coquelle et al., 2010). As observed for non-halophilic counterparts, this halophilic enzyme does not require salt to maintain its conformational stability. However, the *Sr* MalDH structure revealed an acidic amino acids enriched surface, typical to that observed for a halophilic enzyme, which is responsible for a favorable change of solubility in high concentration of salts (Coquelle et al., 2010). According to the solvation-stabilization model for halophilic protein (Madern et al., 2000; reviewed in Zaccai, 2013), high salt concentrations exert a major selective pressure through a strong impact on protein solubility. In order to compete against this deleterious effect of salts, halophilic proteins stay highly soluble by maintaining a solvation envelope composition as close as possible as the composition of the bulk. This model is based on biophysical measurements that have shown that a halophilic protein recruits a solvation envelope of high ionic concentration (Costenaro et al., 2002; Ebel et al., 2002). In the solvation-stabilization model, surface acidic amino acids are suggested to be responsible for this particular solvent organization. Even if several structure of halophilic protein have been solved (Frolow et al., 1996; Richard et al., 2000; Bieger et al., 2003; Irimia et al., 2003; Zeth et al., 2004; Besir et al., 2005; Britton et al., 2006; Winter et al., 2009; Yamamura et al., 2009; Wende et al., 2010; Bracken et al., 2011), attempts to describe how the solvation shell of a halophilic protein interacts with acidic residues using X-rays crystallography is still a challenge.

In our follow-up crystallographic study on *Sr* MalDH (Coquelle et al., 2010); we determined the direct effect on solvent organization due to its acidic surface, by using a comparison with a non-halophilic counterpart. For this purpose, we solved *de novo* the crystal structure of the non-halophilic *Chloroflexus aurantiacus* (*Ca*) MalDH at 1.7 Å resolution. It allowed the determination of a hydration shell consisting in 945 water molecules, which cluster themselves in large networks of structured water through pentameric/hexameric polygons. Direct and indirect effects of acidic amino acids substitutions, avoiding the formation of structured water in *Sr* MalDH are described here through the comparison with *Ca* MalDH. The data are analyzed with respect to the solvation-stabilization model for halophilic protein. In particular, we underline that difference in hydration-solvation characteristics should always be kept in mind while analyzing the solvation layer of a halophilic protein, using X-ray crystallography, or any other techniques.

## Materials and methods

### Protein production and purification

*Ca* MalDH overexpression was done accordingly to Dalhus et al. (2002). The cells were lysed by sonication in a 50 mM Tris-HCl buffered at pH 7. The crude extract was incubated for half an hour at 70°C and centrifugated for 15 min at 17,000 g. The soluble portion of the extract was loaded on a Q sepharose column equilibrated in 50 mM Tris-HCl buffer at pH 7. The protein was eluted using a linear gradient of 0–1 M NaCl. Fractions containing *Ca* MalDH were extensively dialyzed against 50 mM potassium phosphate buffer (pH 7) and deposited on a hydroxyapatite column equilibrated with the same buffer. The enzyme was eluted with a linear gradient of 50–1000 mM ammonium phosphate. The active fractions were pooled and concentrated by centrifugation using an Amicon PM30. They were deposited on a Sephacryl S300 gel filtration column (1 × 100 cm) and then eluted using an isocratic buffer of 50 mM Tris-HCl buffered at pH 7. The purified fractions were concentrated at 20 mg/ml and stored at 4°C.

### Crystallization

Crystallization was performed by vapor diffusion using the hanging-drop method at 293 K. Native *Ca* MalDH crystals (≈500 × 400 × 400 μm^3^) were grown within 2 days by mixing 1.5 μL of 20 mg· mL^−1^ protein solution and 1.5 μL of 4–14% PEG 400, 100 mM sodium acetate buffer at pH 4.6 and 40 mM cadmium acetate reservoir solution. *Ca* MalDH derivative crystals were obtained by a 10 s soaking of a native crystal in a 2.0 μL solution equivalent to the mother liquor containing 100 mM of GdHPDO3A lanthanide complex (Girard et al., 2003). Then the crystal was quickly back-soaked in 2.0 μL of the corresponding reservoir solution without the lanthanide complex.

Prior to data collection, native and derivative crystals were cryo-cooled in liquid nitrogen using mother liquor containing 25% PEG 400 as cryo-protectant.

### Data collection and data processing

Gd-derivative data were collected on a Nonius FR591 X-Ray home source (1.541 Å). Native data were collected on the FIP-BM30A beamline at the ESRF (Grenoble, France) with the X-ray beam wavelength set to 0.979 Å. Diffraction frames were integrated using the program XDS (Kabsch, 2010) and the integrated intensities were scaled and merged using the CCP4 programs SCALA and TRUNCATE (Winn et al., 2011) respectively. A summary of the processing statistics is given in Table 1.

**Table 1:** **Data collection and processing statistics**.

	**Data set**	
	**GdHPDO3A derivative**	**Native**
λ (Å)	1.541	0.979
Space group	P3121	
Cell parameter (Å)	a = 106.77, c = 103.53	a = 106.23, c = 102.57
Resolution (Å)	19.63–1.90 (2.00–1.90)	19.70–1.70 (1.79–1.70)
Unique reflexions	54120 (7772)	68673 (10178)
*R*_merge_ (%)^a^	5.0 (23.7)	8.4 (35.6)
*R*_pim_(%)^b^	1.9 (8.5)	4.4 (20.3)
*R*_ano_ (%)^c^	3.6 (9.6)	
I/I(σ) (d)	12.4 (3.3)	6.3 (2.2)
Completeness (%)	98.8 (99.1)	93.7 (95.8)
Multiplicity	10.6 (9.8)	4.2 (3.9)

^a^*R*_merge_ = $\sum_{h}\sum_{i}\left|\bar{I}(h)-I_{i}(h)\right|/\sum_{h}\sum_{i}\left|I_{i}(h)\right|$ where *I*_*i (h)* is the ith measurement of reflection h and Ī*(h)* is the mean measurement of reflection h.

^b^R_p.i.m._ = $\sum_{h}{\left(\frac{1}{\left(N-1\right)}\right)}^{1/2}\sum_{i}\left|I_{i}(h)-\bar{I}(h)\right|/\sum_{h}\sum_{i}I_{i}(h)$. This indicator, which describes the precision of the averaged measurement, is most relevant. (Weiss, 2001).

^c^R_ano_ = $\sum_{h}\left|{\bar{I}}^{+}(h)-{\bar{I}}^{-}(h)\right|/\sum_{h}\left|{\bar{I}}^{+}(h)+{\bar{I}}^{-}(h)\right|$ where Ī^+^ (h) and Ī^−^ (h) are the mean intensities of a Friedel mate.

^d^*I*σ*(I)* is the signal-to-noise ratio for merged intensities.

*Ca* MalDH crystals belong to the P3_1_21 space group with one A-D dimer per asymmetric unit leading to a solvent content of 49.5%.

### Experimental siras phasing

*Ca* MalDH structure was determined *de novo* by the SIRAS (Single Isomorphous Replacement with Anomalous Scattering) method. As shown in Table 1, the high value of Rano clearly indicated the presence of GdHPDO3A complex binding sites, which was then confirmed by inspection of the anomalous Patterson map. Gadolinium positions were determined within the asymmetric unit using the program SHELXD (Sheldrick, 2010). Heavy-atom refinement and initial phasing were performed using the program SHARP (Bricogne et al., 2003). Phases from SHARP were improved by density modification using the CCP4 program DM (Cowtan and Main, 1996) leading to figures of merit of 0.235 and 0.793 after SHARP and density modification respectively. Automatic model building was performed with the program BUCCANEER (Cowtan, 2006) leading to an initial model consisting in 552 over the expected 618 A-D dimer residues.

### Refinement and water molecules building

The model was manually completed and improved in COOT (Emsley et al., 2010) prior to refinement with PHENIX (Adams et al., 2010). This model was then optimized through iterative rounds of refinement and model building. At the end stages of the refinement, TLS was used with TLS-groups determined with the TLSMD server (Brünger, 1992; Painter and Merritt, 2006a,b). The 1.7 Å resolution *Ca* MalDH final model consists in the complete (N-terminus, C-terminus and catalytic loop) residues sequence for each monomer of the *Ca* MalDH A-D dimer. The analysis of this final model (Table 2) showed no residues in disallowed regions of the Ramachandran plot (99.7% in preferred regions).

**Table 2:** **Refinement statistics and model quality of the *Ca* MalDH structure**.

**PDB Code**	**4CL3**
Resolution (Å)	19.70–1.70
Rwork (%)^a^	17.02
Rfree (%)	21.01
Number of reflexion used	67,827
**ATOMIC COMPOSITION**	
Protein	4694
Water	945
Ions	12
Ligands	55
Res.out of Ramachandran (%)	0.30
**GLOBAL STANDARD DEVIATION**	
Bond length (Å)	0.010
Bond angle (°)	1.292
**BFACTOR VALUES**	
Mean protein Bfactor (Å^2^)	21.50
Min protein Bfactor (Å^2^)	9.17
Max protein Bfactor (Å^2^)	109.92
Mean water Bfactor (Å^2^)	36.04
Mean ions Bfactor (Å^2^)	34.55
Mean ligand Bfactor (Å^2^)	32.47

^a^*R*= $\sum_{h}\left|F_{o}-F_{c}\right|/\sum_{h}\left|F_{o}\right|$ where *F_o_* and *F_c_* are the observed and calculated structure factor amplitudes of reflection h respectively. Rfree (Brünger, 1992) is the R for the test reflection data set for cross validation (5% of excluded reflections). Rwork is the R for the working reflection data set.

In order to precisely assign the 945 water molecules in the model, we allowed the PHENIX program to automatically build solvent molecule up to 5.0 Å above the protein surface, with a distance of 1.7–3.0 Å between two water molecules or between a water molecules and the coordinated residue and only if the 1.0 σ contored 2*F*_o_−F_c_ electron density map was interpretable. At the end, each water molecule was manually verified in COOT.

All the figures were made by using the Pymol program: The PyMOL Molecular Graphics System, Version 1.5.0.4 Schrödinger, LLC. All electrostatic calculations were performed using the Pymol plugin for APBS (Baker et al., 2001).

## Results

### Quality of *Ca* and *Sr* MalDH models

**Figure 1:**
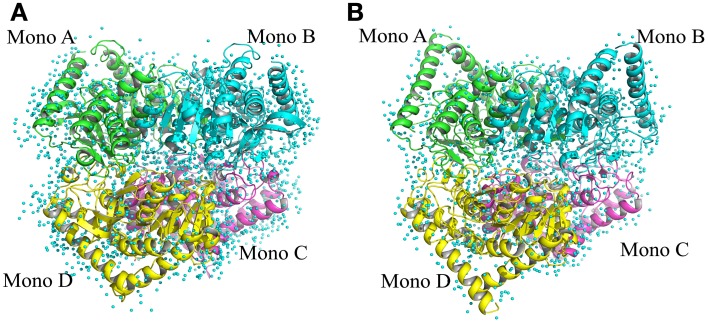
**Ribbon drawing of tetrameric MalDH. (A)** *Chloroflexus aurantiacus* MalDH. **(B)** *Salinibacter ruber* MalDH. Monomers are represented in four different colors. The water molecules are displayed by small blue spheres.

The structure of *Ca* MalDH enzyme was determined at 1.7 Å resolution using SIRAS phasing. The asymmetric unit contains a dimer, the physiological tetramer being generated by the crystal symmetry operators of the P3_1_21 space group (Figure 1). This dimer delineated A-D will serve as the reference for all comparisons through this study. Our *Ca* MalDH model (4BGT) does not present major fold difference compared to the previously deposited (PDB accession code: 1GUY) structure (Dalhus et al., 2002), as confirmed by a root-mean-square deviation (RMSD) value of 0.42 Å for 594 A-D dimer superimposed residues. Moreover, the mobile loop (residues 83–89, following the linear numbering of 4BGT) covering the catalytic site, as well as the residues of the N- and C- termini have been modeled in each monomer of this new *Ca* MalDH structure. The detailed analysis of *Ca* MalDH fold and stabilization mechanism based on the 1GUY model has previously been published (Dalhus et al., 2002), and thus will not be further described in this study. The striking new feature in our model is the incredibly large number of modeled water molecules, i.e., 945 for the dimer A-D, which allows a detailed analysis of water organization.

*Sr* MalDH shares more than 72% of sequence similarity with its non-halophilic counterpart *Ca* MalDH. The *Sr* MalDH model was obtained at a resolution of 1.55 Å, and also contains a large number of water molecules: 680 for the equivalent *Ca* MalDH A-D dimer (Coquelle et al., 2010). The overall structural similarity between one monomer of *Sr* and *Ca* MalDHs led to a RMSD of about 0.6 Å for 258 superimposed Cα.

Therefore, these two structures of excellent resolution, with a large number of water molecules in their solvent layers, provide a unique combination to finely compare the water organization at their surface.

### Comparison of halophilic and non-halophilic hydration patterns

**Figure 2:**
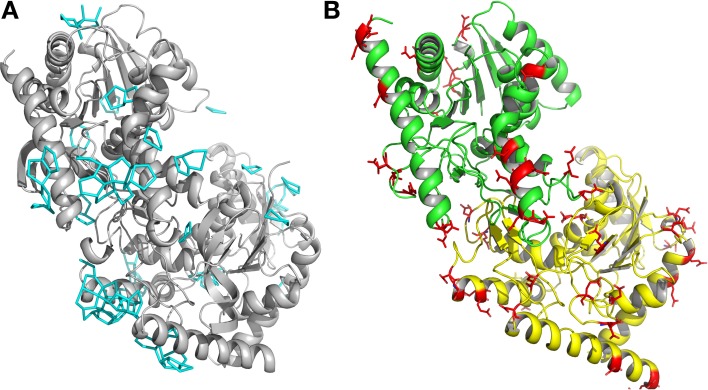
**(A)** Ribbon drawing of monomers A and D of *Ca* MalDH. The water polygons are represented in blue lines. **(B)** Ribbon drawing of monomer A (green) and monomer D (yellow) of *Sr* MalDH. The surface acidic amino acid is shown in red.

A detailed analysis of the geometry of the 945 water molecules surrounding the dimeric *Ca* MalDH model (distance and angle) was performed and is presented in Table 3. It is outside the scope of this study to describe in great details both the geometry and interactions with the protein of all these water molecules. The role of water molecules in the folding process and stabilization of proteins has been well described in a work based on a larger set of proteins (Matsuoka and Nakasako, 2009). The most interesting feature of the water molecules in *Ca* MalDH structure is that 28% of them are organized in polygons (pentagons or hexagons), which can form extended clusters (Figure 2). These polygons are only observed at the surface of apolar residues. Geometrical properties of these polygons (Table 3) are in good agreement with those determined from a large statistical study using high-resolution structures (Lee and Kim, 2009).

**Table 3:** **Water statistics for dimer AD of *Ca* MalDH**.

Number of water molecules: 945		
Water per residues:1.57		
Water molecules involved in polygons: 28%		
76 polygons: 10 hexagons and 66 pentagons		
Size of clustered polygons: Up to 15		
Planar polygons: 64%		
Distance between surface residues and polygons (in Å)		
Minimal 2.58	Maximal 4.02	Average 3.23
Distance between two water molecules forming pentagons (in Å)		
Minimal 2.11	Maximal 4.19	Average 2.86
Angle between three water molecules forming pentagons (in °)		
Minimal 73.86	Maximal 138.74	Average 107.56
Distance between two water molecules forming hexagons (in Å)		
Minimal 2.39	Maximal 3.32	Average 2.66
Angle between three water molecules forming hexagons (in °)		
Minimal 81.45	Maximal 142.52	Average 111.72

**Figure 3:**
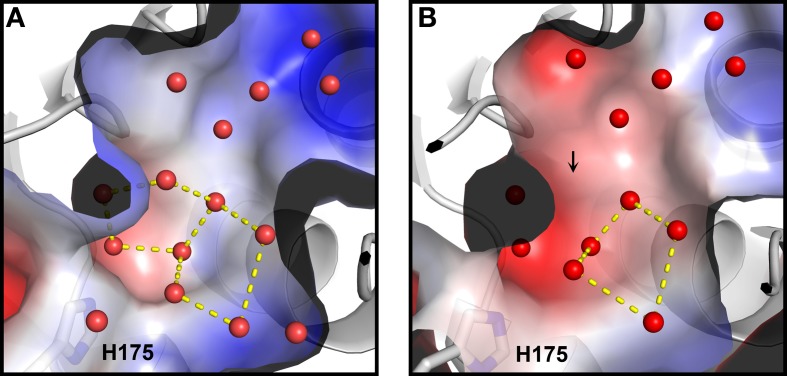
**Close up views of the catalytic pocket**. Electrostatic surface representation of *Ca* MalDH **(A)** and *Sr* MalDH **(B)**. Water molecules are shown in small red spheres. Dashed lines cultured in yellow delineates the polygons. The catalytic histidine (H175) is indicated. Numbering of amino acids corresponds to linear numbering of *Ca* MalDH.

Based on *Ca* MalDH water analysis, a careful inspection of the halophilic *Sr* MalDH hydration layer at the surface of the protein was performed to detect any water polygon. Even though 43% of *Sr* MalDH water molecules were considered to be superimposable with those from *Ca* MalDH (using a cut off distance of 1.5 Å), no polygons were observed at the surface of the halophilic MalDH. However, 14 water molecules lie in the catalytic pocket of *Sr* MalDH, all of which are conserved in *Ca* MalDH. Five are organized as a pentagon, the only one observed in *Sr* MalDH (Figure 3). In *Ca* MalDH, the same water pentagon is present, but the catalytic pocket of *Ca* MalDH contains an extra water molecule, which closes a second pentagon in the catalytic pocket, adjacent to the first one (Figure 3A). A black arrow indicates the missing water molecule in *Sr* MalDH (Figure 3B).

We therefore decided to have a closer look at surface regions where polygons are present in *Ca* MalDH to figure out the reasons why none are observed in *Sr* MalDH.

### Acidic *Sr* MalDH surface prevents the formation of structured water

**Figure 4:**
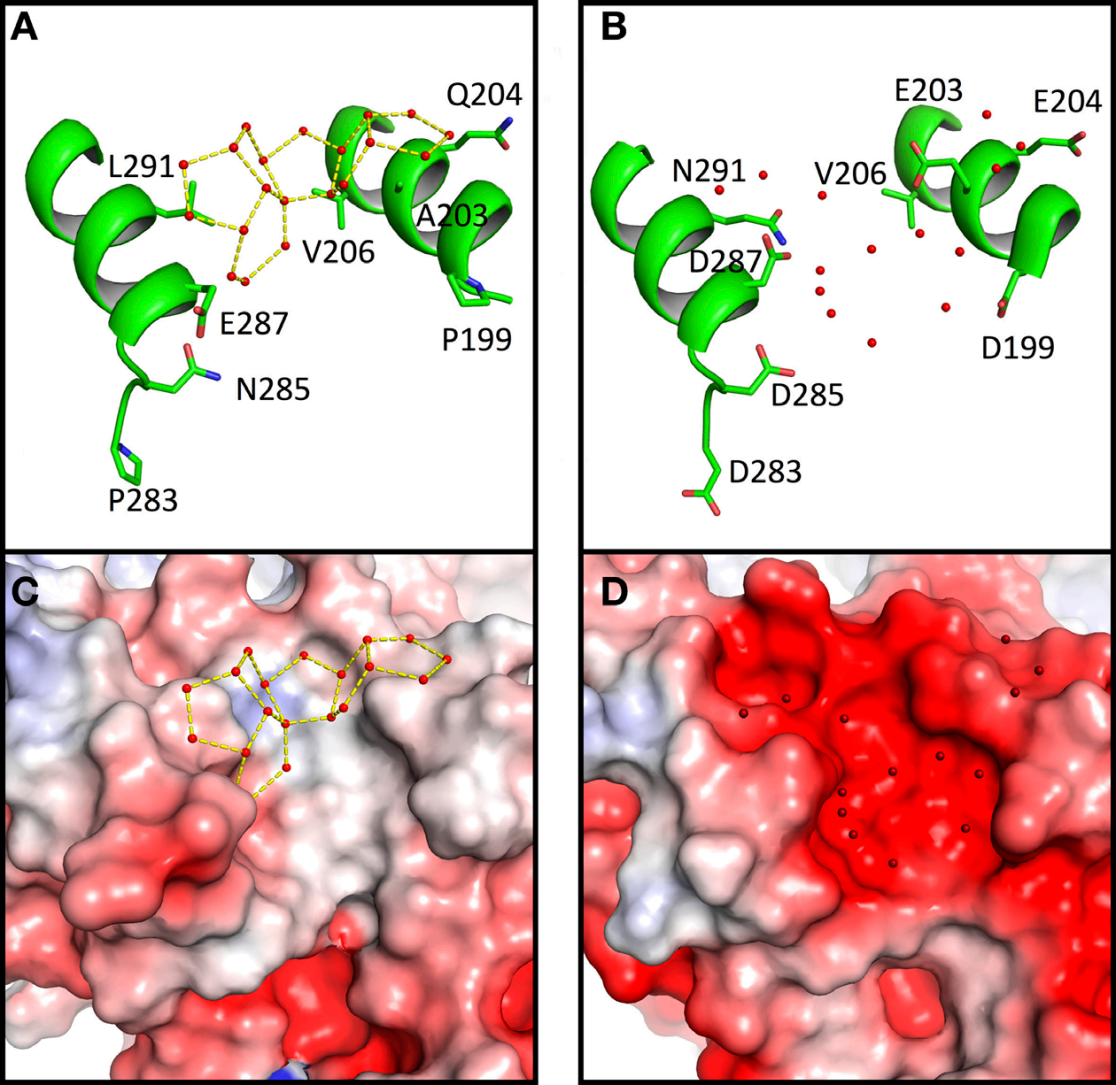
**Close up views of *Ca* MalDH **(A)** and *Sr* MalDH (B)**. Water molecules are shown in small red spheres. Dashed lines coloured in yellow delineates the polygons. Important amino acids are represented in sticks. Electrostatic surface representation of *Ca* MalDH **(C)** and *Sr* MalDH **(D)**.

As mentioned, large networks of connected water polygons are present in *Ca* MalDH (Figure 2A). An example of such network is shown in Figure 4A. This network is anchored between helices α1G-α1G and αH and is made up of five pentagons and one hexagon. In the same protein region, no water polygon is observed in *Sr* MalDH (Figure 4B), which possesses four extra negative charges compared to *Ca* MalDH, due to substitutions at positions 199, 203, 283, and 285. These substitutions led to important electrostatic surface changes, with a highly negative one for *Sr* MalDH compared to the apolar surface of *Ca* MalDH (Figures 4C,D). The lateral chain of acidic residues D287 in *Sr* MalDH is orientated in such a conformation that the *Sr* MalDH hydration pattern is modified when compared to that of *Ca* MalDH. The data suggest that the replacement of non-polar amino acid residues by acidic amino acid in a halophilic protein modifies properties of the hydration shell. Around apolar surfaces of the non-halophilic MalDH, water molecules cannot form direct hydrogen bonds with the protein, and thus organize themselves as polygons with their nearest stable water neighbors. Acidic amino acids enrichment in these regions of *Sr* MalDH surface favors direct hydrogen bonding with water and therefore prevents polygons formation.

**Figure 5:**
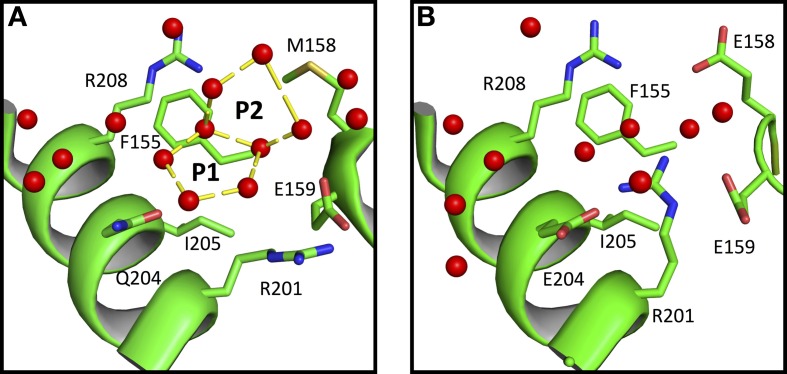
**Close up view of *Ca* MalDH (A) and *Sr* MalDH (B)**. Water molecules are in red spheres. Linear numbering as in *Ca* MalDH.

We also observe that water polygons formation is hampered in halophilic *Sr* MalDH, not only by direct acidic amino acid substitution but also by the side chain reorganization of conserved residues, as illustrated in Figure 5. In *Sr* MalDH compared to *Ca* MalDH, two acidic amino acids are observed at position 158 and 204. Glutamate at position 158 induces a direct perturbation of water pentagon P1, as previously observed. But Glutamate 204 promotes an interaction with R201 side chain, which moved to a new position that hinders appropriate hydrogen bonding geometries requested for the formation of water polygon P2 (Figure 5).

These two examples clearly illustrate the key influence of acidic amino acid enrichment in halophilic protein on the water organization at their surface; either through direct impacts or *via* conformational rearrangements of surrounding residues. This leads to the destabilization of almost all water polygons observed in the non-halophilic protein structure.

## Discussion

This study presents for the first time a detailed analysis of the water organization at the surface of a halophilic protein and its non-halophilic counterpart. Both crystal structures were obtained at high resolution (better than 1.7 Å) and display similar crystallographic quality. The comparison of these hydration envelopes shows the effect of surface composition changes on the hydration shell structure. In the structure of *Ca* MalDH, we observed a large amount of stable water polygons. These specific water arrangements were first observed in the crystal structure of Crambin (Teeter, 1984). It has been analyzed that these water are not the results of crystallization process and are likely due to intrinsic interaction mode with the local hydrophobic surface of proteins (Nakasako, 1999, 2004). Water organization observed in *Ca* MalDH is in good agreement as apolar surface prevent direct hydrogen bonding of water molecules with the protein and favors polygonal structures. Acidic residues substitutions at the surface of *Sr* MalDH promote hydrogen bonding between the solvent and the protein. In particular, we observed that the changes in water structure organization in *Sr* MalDH are not only due to direct effects but also to long-range effects of amino acid substitutions. The latter is an indirect consequence of amino acids substitutions, selected to increase the *Sr* MalDH enzymatic activity at high salt concentration as analyzed in our previous work (Coquelle et al., 2010). Indeed, these changes modify the local dynamics of the protein surface, which should impact the dynamical properties of the nearest hydration water molecules, as previously observed (Nakasako et al., 2001).

At this stage, it is important to remind the concept of solvation/hydration of proteins.

### Are halophilic proteins solvated or hydrated?

**Figure 6:**
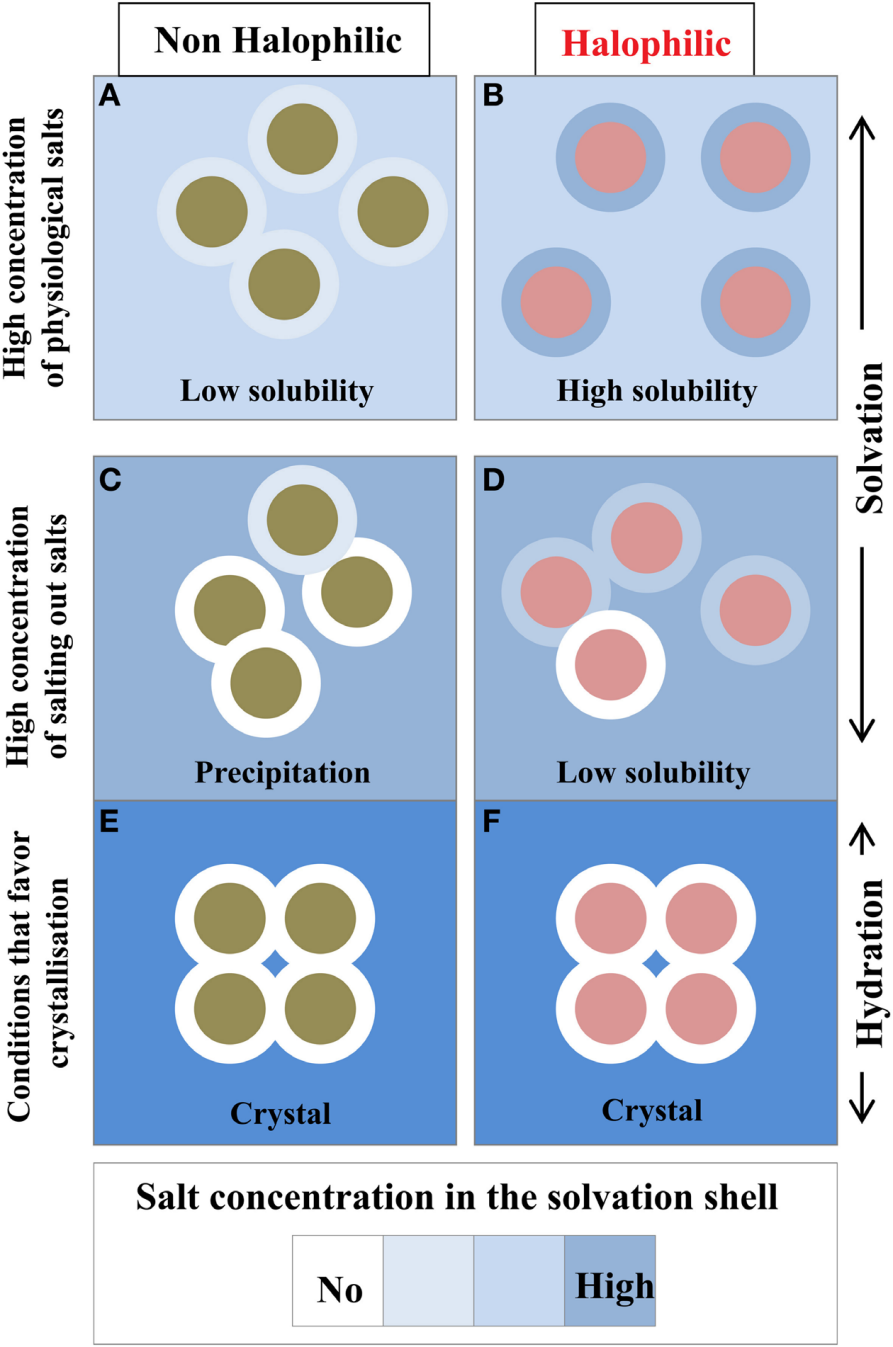
**Compositional changes of the solvation layer between non-halophilic and halophilic proteins**. Filled circles either represent non-halophilic (Green) or halophilic (Red) proteins with their solvation shell (external circle). Solubility measurements are taken from Coquelle et al. (2010). With the halophilic protein, due to its acidic enriched surface, the dominant inter particular effect is repulsive and its solvation shell composition is similar to the bulk solvent **(B)**. These two effects strongly favor high solubility even in high physiological salt concentration. In equivalent conditions the non-halophilic protein solubility is reduced **(A)**. In non-physiological conditions, i.e., in the presence of salting out salts **(C,D)** or additives that promote crystallization **(E,F)**, the solvation shell of each enzyme starts to be depleted in salt. This situation impacts the solubility of each enzyme to diverse extent and could promote precipitation. In crystal conditions, the solvation envelope is a hydration shell, which contains none or very few ions.

This is an important issue that should be discussed. Because of the chemical properties of the protein surface, the solvent composition at the vicinity of a given protein surface is different from the bulk. In a simple binary system containing water and protein without any cosolvents, such as salt or other macromolecular solutes, a hydration shell surrounds the protein. In the presence of high concentration of additional compounds such as salts, sugars, precipitating agents etc., the protein solution should be described as a ternary system in which the protein is enveloped by a solvation shell. The thermodynamics of proteins in the three-component system is well understood in terms of preferential binding parameters (Von Hippel and Schleich, 1969; Inoue and Timasheff, 1972; Arakawa and Timasheff, 1982; Zaccai and Eisenberg, 1990; Timasheff, 1991; reviewed in Zaccai, 2013). In conditions that maintain protein solubility, the chemical potential of the solvation shell and the bulk are equilibrated (Figure 6). In salting-out conditions that favor protein aggregation and crystallization, the equilibrium is strongly perturbed because the small solutes are excluded from the solvation shell (Tardieu et al., 2002). In this case the solvation shell looks like a hydration shell.

Cytoplasmic protein isolated from extreme halophilic prokaryotes that use the KCl-in adaptive strategy, such as *S. ruber* or the *Halobacteriaceae*, maintain a high solubility at molar concentration of various salt (Coquelle et al., 2010). In the case of the tetrameric MalDH from *Haloarcula marismortui*, the measurements of the preferential binding parameters have shown that the enzyme obey the general thermodynamics rules of the three components system (Costenaro et al., 2002; Ebel et al., 2002): In salting out conditions, the solvation envelope of *Hm* MalDH is strongly depleted in salt and it looks like a hydration shell; such behaviors is equivalent to the situation encountered with a non-halophilic protein. However, in high concentration of various physiological salts, it has been measured that *Hm* MalDH preferential binding parameters depend on salt type, demonstrating that the composition of its solvation shell varies (Costenaro et al., 2002; Ebel et al., 2002). In these physiological salts, *Hm* MalDH solvation envelope is enriched in salt, reflecting its halophilic adaptation. Consequently, as the chemical potential of the solvation layer and the bulk solvent are close, *Hm* MalDH remains highly soluble at high salt concentration. We determined that *Sr* MalDH remains highly soluble in high concentration of physiological salts (Coquelle et al., 2010). Based on the observation made on *Hm* MalDH, this suggests that *Sr* MalDH solvation layer should also be enriched with salts.

### Solubility and acidic amino acid surface enrichment

The relationship between an increase in protein solubility and the shift toward negatively charged protein surfaces is not restricted to halophilic protein (Trevino et al., 2007). The favorable effect of acidic residues on protein solubility has been highlighted by an elegant thermodynamical work based on seven non-halophilic proteins, which displayed pIs ranging from 3.5 to 8 (Kramer et al., 2012). In the case of halophilic proteins, it has been demonstrated that their high negative charge density maintains a weak repulsive protein-protein interactions in high salt concentration (Costenaro et al., 2002; Ebel et al., 2002). Theoretically, this repulsive effect between macromolecules of same net charge could also be induced by positively charged amino acids. However, calculation of the solvent-accessible areas of the side-chain components between negatively and positively charged residues unravel their relative efficiency on solubility. Compared to positively charged residues, the favorable effect of acidic residues is due to the lower hydrophobic solvent exposed surface of their side chain (Britton et al., 1998).

### Application to halophilic protein structures

Our comparative study of the hydration shell of *Ca* and *Sr* MalDHs sheds light on the close relation between solubility, acidic residue enrichment and solvation.

First, conclusions from the solvent properties analysis using X-ray crystallography should be drawn with cautiousness. Our observation confirms that salting-out conditions deplete the solvation layer in salts. Indeed, the additives used for crystal growth shift the conditions toward salt depletion in the solvation envelope. Consequently, even if several halophilic proteins have been crystallized in the presence of high concentrations of physiological salts, one should take precaution when discussing the role of the solvent layer as obtained from X-ray structures.

Second, the favorable change in solubility of halophilic proteins is driven by their protein surface enrichment in acidic residues, which plays a dual role. Indeed, our study shows that acidic residues, through their carboxyl groups that are known to form strong hydrogen bonds, can organize the solvation shell by direct as well as indirect interactions. They are therefore good candidates for interactions with hydrated salt ions as proposed by Zaccai (2013). Moreover, they promote slightly repulsive inter-particular interactions between each protein molecule, favoring solubility.

## Conclusion

Recent data have suggested that acidic enrichment, considered as an adaptive signature of halophilic proteins, could also be due to genetic drift (Deole et al., 2013). Whatever the precise evolutionary mechanism responsible for the peculiar composition of protein isolated from halophilic microorganisms, our work helps to understand that acidic acid enrichment was an appropriate evolutionary innovation in the case of microorganisms that accumulates high concentration of KCl in their cytoplasm to maintain their turgor pressure in highly salted environment. Such enrichment allows halophilic proteins to compete against aggregation via their ability to reorganize protein-solvent interactions.

The role of acidic amino acids substitution on the solvent organization, highlighted in the present work, has to be completed by further studies involving enzymes from halophilic organisms that used different strategies to cope with high concentration of salts.

## Author contributions

Dominique Madern and Eric Girard designed research, Romain Talon, Nicolas Coquelle, Dominique Madern, and Eric Girard performed research, Romain Talon, Nicolas Coquelle, Dominique Madern, and Eric Girard were involved in data analysis. Romain Talon, Nicolas Coquelle, Dominique Madern, and Eric Girard wrote the paper.

### Conflict of interest statement

The authors declare that the research was conducted in the absence of any commercial or financial relationships that could be construed as a potential conflict of interest.
